# The development and transcriptome regulation of the secondary trunk of *Ginkgo biloba* L.

**DOI:** 10.3389/fpls.2023.1161693

**Published:** 2023-05-30

**Authors:** Zhong-yun Cao, Li-ning Su, Qian Zhang, Xin-yue Zhang, Xiao-jing Kang, Xin-hui Li, Li-min Sun

**Affiliations:** ^1^ State Forestry and Grassland Administration Key Laboratory of Silviculture in Downstream Areas of the Yellow River, Forestry College of Shandong Agricultural University, Tai’an, Shandong, China; ^2^ Shandong Academy of Forestry Sciences, Jinan, Shandong, China

**Keywords:** *Ginkgo biloba*, secondary trunk, anatomy, endogenous hormones, transcriptome sequencing

## Abstract

Secondary trunk *Ginkgo biloba* is one of the specific germplasms of *G. biloba*. In this study, paraffin sectioning, high-performance liquid chromatography and transcriptome sequencing technology were used to study the development of the secondary trunk of *G. biloba* from the morphological, physiological and molecular levels. The results showed that the secondary trunk of *G. biloba* originated from the latent buds in the stem cortex at the junction of the root and stem of the main trunk. The development process of secondary trunk was divided into 4 periods: the dormancy period of the secondary trunk buds, the differentiation period, the formation period of transport tissue, and the budding period. Transcriptome sequencing was performed by comparing the germination period and elongation growth period of the secondary trunk with the normal parts of the same period where no secondary trunks occurred. Differential genes involved in phytohormone signal transduction, phenylpropane biosynthesis, phenylalanine metabolism, glycolysis and other pathways can regulate not only the inhibition of early dormant buds but also the later development of the secondary trunk. Genes related to IAA synthesis are upregulated and indole-3-acetic acid content is increased, leading to the up-regulated expression of IAA intracellular vector genes. The IAA response gene (SAUR) receives and responds to IAA signals to promote the development of the secondary trunk. Through the enrichment of differential genes and functional annotations, a key regulatory pathway map for the occurrence of the secondary trunk of *G. biloba* was sorted out.

## 1 Introduction

*Ginkgo biloba* L. is the only remaining species of the Ginkgoaceae in China during the Quaternary glacial period with high adaptability, longevity and ornamental value (Seward, 1938; Cao, 2007). *G. biloba* is different from other gymnosperm species in that the phenomenon of ‘arising branches from the base of a stem’ (secondary trunk) is common from young trees to thousand-year-old trees. Del Tredici found in his survey of the West Tianmu Mountain Nature Reserve that 40% of the *G. biloba* in the area could produce sprouts, most of which were connected to a callus-like tumor (basal tree tumor) at the base of the stem. Therefore, it was believed that *G. biloba* could be regenerated by secondary trunk from the basal tree tumors, but the origin of the above-ground sprouts was not mentioned (Tredici, 1992a; Tredici, 1992b).

Xing Shiyan named the ‘branches arising from the base of a stem’ of *G. biloba* as the ‘secondary trunk’ for the first time and showed that the secondary trunk of *G. biloba* originated from the latent buds of the stem at the junction of root and stem. And the secondary trunk was essentially different from the root tillers of other tree species. The root system of return-young *G. biloba* does not produce rootstocks like the paulownia and other species. The secondary trunks of *G. biloba* usually grow upright around the main stem, with thick stems, small angles to the main stem, large, thick, multi-lobed leaves, obvious ‘return-young’ characteristics, growing faster than the main stem (Xing, 1996; Xing, 2013). The mechanism of the secondary trunk is related to the type of stem (branch) differentiation system, and the secondary trunk of *G. biloba* was part of the normal development of individuals in the natural state. With the increase of age, the number of secondary trunks increases greatly after the senescence of the tree top or destruction of the top buds of the secondary trunk, and the secondary trunk can be regenerated on the secondary trunk. As the number of secondary trunks increases, the base of the secondary trunk can form root discs. The secondary trunk retains the characteristics of the main stem and has a low basal rooting rate (Xing, 1996). The current research on the secondary trunk of *G. biloba* has mainly focused on the growth characteristics, distribution, regeneration, and utilization of the secondary trunk, but little has been reported on the origin of the secondary trunk of *G. biloba* at the anatomical level and the intrinsic molecular mechanism affecting the development of the secondary trunk.

In this study, the paraffin sectioning method was used to observe the initiation of the secondary trunk of *G. biloba*. By high-performance liquid chromatography, the endogenous hormones were measured in two different developmental stages of the secondary trunk of *G. biloba* and lateral branches to understand the influence of endogenous hormones on the development of the secondary trunk. At the same time, transcriptome sequencing and bioinformatics analysis were used to screen the differentially expressed genes affecting the formation and development of the secondary trunk and to speculate possible transcriptional regulation relationships. Through the research on the development of the secondary trunk, the occurrence mechanism of the secondary trunk can be understood and it can lay a theoretical foundation for revealing the mystery of the longevity of ancient ginkgo trees.

## 2 Materials and methods

### 2.1 Experimental material

The experimental materials for anatomical observation and endogenous hormone determination of the secondary trunk of *G. biloba* were collected from 4 years of seedling *G. biloba* and grafted *G. biloba* in the Forestry Experimental Station (N36°10’, E117°10’) of the South Campus of Shandong Agricultural University. The seedlings grow robustly and free from pests and diseases. The region is located in the southeast of Tai’an City, Shandong Province. It belongs to a warm temperate zone and semi-humid continental monsoon climate.

**Figure 1:**
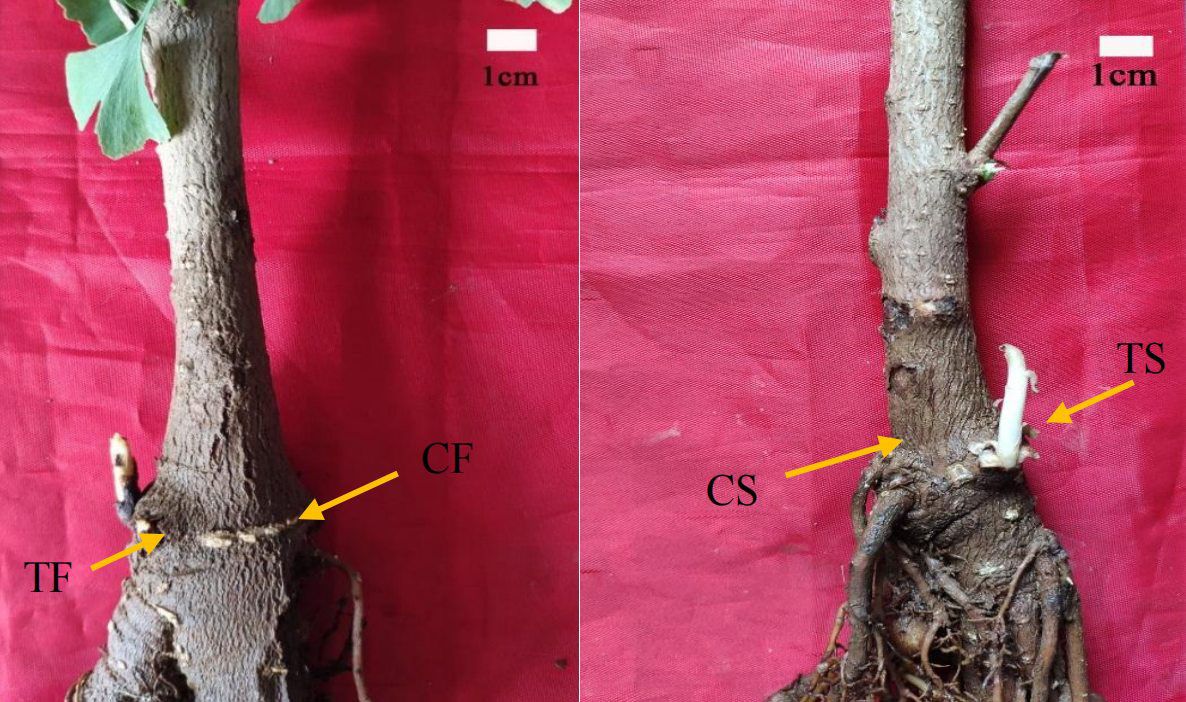
Experimental samples of transcriptome. TF, Secondary trunk at stage of germination; TS, Secondary trunk at stage of elongation; CF、CS, Normal tissue around secondary trunk.

The experimental materials for the transcriptome sequencing of the secondary trunks were collected from the 4-year-old *G. biloba* seedlings from the 75# family of Shandong Forestry Germplasm Resource Center. The seedlings grew robustly and free from pests and diseases (**Figure 1**). The region is located in Zhangqiu District, Jinan City, Shandong Province. It belongs to a warm temperate zone and semi-humid continental monsoon climate.

### 2.2 Experimental method

#### 2.2.1 Anatomical observation on the origin of the secondary trunk of *G. biloba*

For the 4-year-old seedling *G. biloba*, to observe the morphology of dormant buds of the secondary trunk and the development process of the secondary trunk after breaking the dormancy, the following experiment method was used: the *G. biloba* seedlings with consistent growth were divided into 8 groups of 3 replicates each, with 10 seedlings in each replicate. At the end of February, the first sampling was conducted and 5 seedlings were collected from each replicate from the first group to observe the dormant bud status by paraffin section. In early March, the remaining 7 groups of *G. biloba* were treated with stumping before the leaves were unfolded to break the dormant state of buds and promote the occurrence of the secondary trunk of *G. biloba* After cutting, a set of samples were taken at weekly intervals and brought back to the laboratory to make paraffin sections, and stained with safranin and green counterstaining. The paraffin sections were observed and photographed under a Nikon E200 microscope.

#### 2.2.2 Endogenous hormone determination

The secondary trunks and the lowermost lateral branches of four-year-old grafted *G. biloba* were collected at two different developmental stages (germination and elongation), and three replicates of secondary trunks and lateral branches from each developmental stage were set up for the endogenous hormone determination by high-performance liquid chromatography. The specific method referred to Zhang Ning’s extraction and determination method (Zhang, 2019). The hormones determined: zeatin (ZT), auxin (IAA), abscisic acid (ABA), and gibberellin (GA_3_).

#### 2.2.3 Transcriptome sequencing and analysis

The 4-year-old *G. biloba* seedlings from the no.75 family of Shandong Forest Germplasm Resource Center were selected. In April 2018, the secondary trunks of the four-year-old *G. biloba* seedlings of the same family at two stages of development (germination stage and elongation growth stage) were collected, and the normal tissues near the secondary trunk were collected as controls. Three replicates were set up for each period and control samples (**Table 1**). According to the RNA extraction method of Liu Xiaojing (Liu et al, 2018), the total RNA of 12 *G. biloba* samples was extracted to ensure the use of qualified samples for transcriptome sequencing.

**Table 1:** Experimental samples of transcriptome.

sample	repetition	germination stage	elongation growth stage
the secondary trunk	repetition 1	TF1	TS1
	repetition 2	TF2	TS2
	repetition 3	TF3	TS3
contrast	repetition 1	CF1	CS1
	repetition 2	CF2	CS2
	repetition 3	CF3	CS3

After the samples were qualified, 3ug of RNA was taken from each sample as the starting material for library construction. The qualified total RNA samples were enriched into mRNA. The obtained mRNA was broken into short fragments by adding a fragmentation buffer. The fragmented mRNA was then used as a template to synthesize the first strand of cDNA with a six-base random primer. The buffer, DNA polymerase I, RNase H and dNTPs were added to synthesize the second strand of cDNA. The double-stranded cDNA was purified by QiaQuick PCR kit and eluted with EB buffer. The eluted and purified double-stranded cDNA was followed by terminal repair, base A addition and sequencing joint. Finally, different size fragments were selected for PCR amplification to complete the preparation of the library. After the library was constructed, the preliminary quantification was done with Qubit3.0 and the library was diluted to 1ng/ul. Agilent 2100 was used to detect the insert size of the library, and the qRT-PCR method was used to accurately quantify the effective concentration of the library. After the library inspection was qualified, sequencing was carried out by illumine platform.

Raw Data was filtered by removing low-quality sequences and joint pollution to obtain high-quality Clean Data. Mapped Data was get by comparing Clean Data with *G. biloba* reference genome (http://gigadb.org/dataset/100613). DESeq2 was used to analyze differentially expressed genes between the treatment and control groups. Genes with q<0.05 and |log2 Fold change|≥1 were selected as significantly differentially expressed genes, and each comparison group was screened to obtain the number of up-regulated and down-regulated genes. All the different Genes were analyzed by GO (Gene Ontology) and KEGG (Kyoto Encyclopedia of Genes and Genomes). The Sequence data are available in the NCBI Sequence Read Archive (SRA): SRR23730290, SRR23730291, SRR23730292, SRR23730293, SRR23730294, SRR23730288, SRR23730289, SRR23730295, SRR23730296, SRR23730297, SRR23730298, SRR23730299.

The RNA of secondary trunks at two different development stages and normal tissue without the secondary trunk of the same plant at the same location were extracted for reverse transcription, and the expression of differentially expressed genes in the transcriptome was verified. Fifteen randomly selected genes related to the development of the secondary trunk of *G. biloba* were quantified by real-time fluorescence.

## 3 Result

### 3.1 Anatomic characteristics of the origin of the secondary trunk

**Figure 2:**
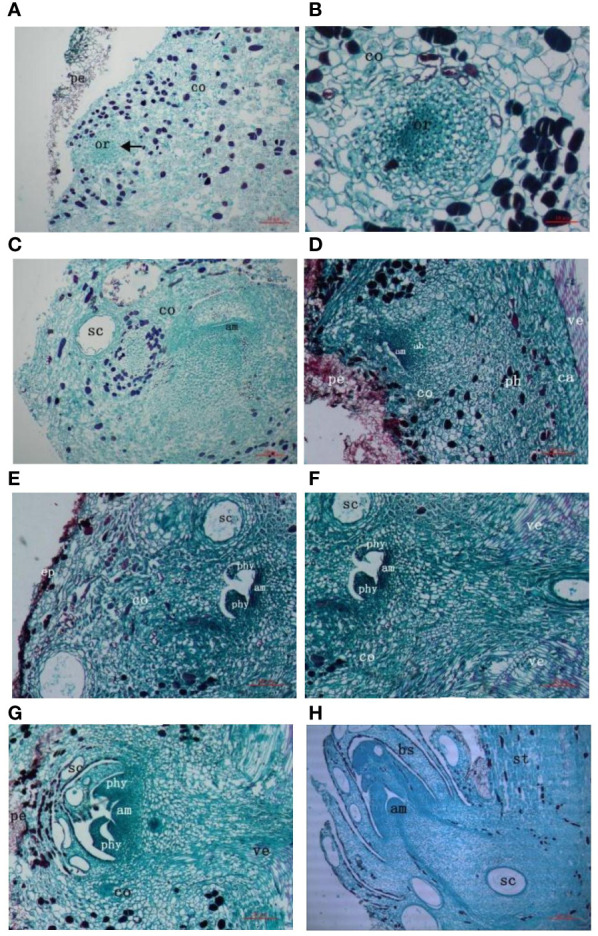
Anatomy of the origin of *G. biloba* secondary trunk. **(A)** The originating cells of secondary trunk form at the cortex. Arrows indicate originating cells. **(B)** The originating cells of secondary trunk differentiate into cell mass. **(C, D)** The cell mass further divides and differentiates to form meristematic regions with cytological division of shoot end. **(E, F)** The meristem region develops into leaf primordium and adventitious bud begins to connect with the vascular system of stem. **(G)** The adventitious buds continue to develop and produce new leaf primordial. The adventitious buds are connected to the vascular system of the stem. **(H)** The buds of secondary trunk form and break bark to grow. Ruler is 10μm. pe, periderm; or, originating cells of secondary trunk, co, cortex; sc, secretory cavity; am, apical meristem; ab, adventitious bud; ph, phloem; ca, cambium; ve, vessel; ep, epidermis; phy, phyllopodium; st, stem; bs, bud scale.

Anatomical studies on the secondary trunk of *G. biloba* at different developmental periods of showed that the development of the secondary trunk can be divided into four periods:

Dormancy period of the buds of the secondary trunk: In the natural state, that was, before the cutting treatment, a longitudinal cut at the base of the *G. biloba* stem showed a group of active parenchyma cells at the cortex, which had a small volume, large nucleus, deep staining, dense arrangement, and relatively vigorous division ability, and formed a densely distributed region. This part of the cells was the germinal cells of the buds of the secondary trunk, namely the dormant buds of the secondary trunk (**Figure 2A**). This part of cells differentiated into a primordial cell group composed of 1-2 layers of cells at the top through vertical and horizontal divisions, and a central blast cell area derived from the primordial cell group was below. The rib-shaped meristem area was under the central blast area. The meristem region was a cluster of closely arranged cells in a sub-circular or oval shape, the cells of the cluster were significantly smaller than surrounding parenchyma cells, and the cells were not uniform in size. One side of the cells were smaller, closely arranged, with a large nucleus, dense cytoplasm and deep staining, and the other side of the cells were larger and sub-circular. The cell cluster showed a strong capacity to divide (**Figure 2B**).

Differentiation period: Series of sections after cutting treatment showed that the dormancy stage of dormant buds was broken and the cell mass began to differentiate further, forming a clear gap above the primitive cell mass, which gradually enlarged. The apical cells in the meristematic region were closely arranged, with dense cytoplasm, deep staining, and vigorous division, forming a slight protrusion in the middle, which was the apical meristem. At the same time, the peripheral meristem areas on both sides of the apical meristem also showed strong splitting ability, but the differentiation of leaf primordium was not obvious (**Figures 2C**, **D**).

The formation period of the transport tissue: With the continuous development of the adventitious buds and the enlargement of the apical gap, the apical meristem formed more obvious protrusions. The surrounding meristem maintained a high frequency of cell division during the development of adventitious buds and formed protrusions after periclinal division, resulting in two leaf primordium. At this time, obvious vascular tissue could be seen at the bottom of adventitious buds, indicating that adventitious buds began to connect with the vascular bundle of the stem, forming a continuous vascular system to ensure that the trunk provided sufficient nutrients for the development of the buds of the secondary trunk (**Figures 2E**, **F**). The leaf primordium continued to grow to form young leaves and the secretory cavity began to exist in the young leaves. The surrounding meristem developed further to create a new leaf primordium on the inner side of the young leaf, at which point a continuous vascular system had been formed (**Figure 2G**).

Germination period: Adventitious buds continued to differentiate and produced new young leaves, with a certain number of secretory cavities in the young leaves, bud scales and buds. Since the tangential division speed of the rib-like meristem was greater than that of the periclinal division, the cells proliferated continuously in the longitudinal direction, so that the adventitious buds broke through the bark of the main trunk and developed into the secondary trunk under suitable light, temperature and moisture conditions (**Figure 2H**).

### 3.2 Hormone changes in the growth process of the secondary trunk and lateral branches

During the germination and elongation growth period, the content of each hormone in the secondary trunk was significantly higher than that in the lateral branches. During the germination period, the content of IAA in the secondary trunk (1.52μg·g^-1^) and the lateral branches (1.47μg·g^-1^) remained at a high level, and the content of IAA in the secondary trunk was significantly higher than that in the lateral branches (t <0.05) at the same development stage. In addition, the content of ZA and GA_3_ in the secondary trunk (0.98μg·g^-1^、1.26μg·g^-1^) was significantly different from that in the lateral branch (0.66μg·g^-1^、0.63μg·g^-1^) (t <0.01). And the content of the two hormones in the secondary trunk was also higher than that in the lateral branch.

The content of various hormones in the secondary trunk also showed significant differences at different developmental stages (t<0.05). At the elongation stage (1.64μg·g^-1^、1.58μg·g^-1^), the content of IAA and ZA in the secondary trunk increased significantly (t<0.05) compared with that at the germination stage (1.52μg·g^-1^、0.98μg·g^-1^), while the content of GA_3_ decreased significantly (t <0.01). The same expression trend was also observed in the lateral branches.

**Figure 3:**
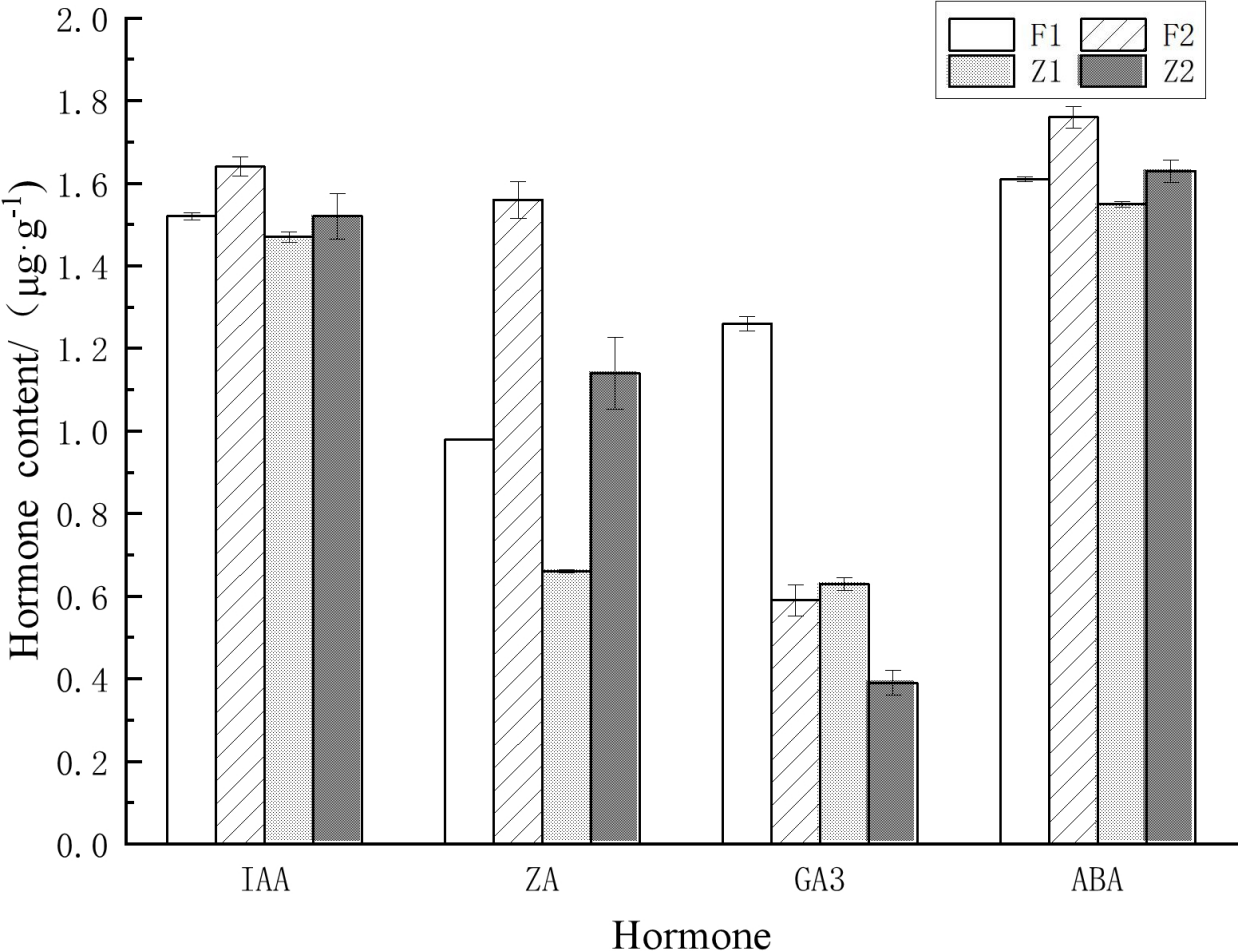
Hormonal changes at different developmental stages in the secondary trunk and the lateral branch of *G. biloba*.

Among the common endogenous hormones in plants, ABA is a growing-inhibiting hormone, while IAA, ZA and GA_3_ are growing-promoting hormones. IAA and ZA synergistically affect the formation and development of buds, and ZA is the determinant of bud germination and growth. Therefore, IAA/ZA and (IAA+ZA+GA_3_)/ABA are the two more referential ratios. The results showed that the ratios of IAA+ZA+GA_3_/ABA were greater than 1 for both the secondary trunk and lateral branches at the germination stage, and the ratio of IAA+ZA+GA_3_/ABA in the secondary trunk was significantly greater than that of the lateral branch (t <0.01), and the ratio of IAA/ZA was significantly smaller than that of the lateral branch (t <0.01). During the elongation growth period, the ratios of IAA+ZA+GA_3_/ABA were greater than 1 for both the secondary trunk and lateral branches, and the ratio of IAA+ZA+GA_3_/ABA in the secondary trunk was greater than that of the lateral branches, while the ratio of IAA/ZA was smaller than that of the lateral branches. The ratio of (IAA+ZA+GA_3_)/ABA decreased during the elongation period compared to the germination period and the growth trend slowed down, while the ratio of IAA+ZA+GA_3_/ABA increased slightly in the lateral branches (**Table 2**; **Figure 3**).

**Table 2:** Hormonal changes at different developmental stages in secondary trunk and lateral branch of *G. biloba*.

Plant organs	IAA μg·g^-1^	ZA μg·g^-1^	GA_3_ μg·g^-1^	ABA μg·g^-1^	IAA/ZA	(IAA+ZA+GA_3_)/ABA
F1	1.52 ± 0.0088	0.98 ± 0.0000	1.26 ± 0.0176	1.61 ± 0.0058	1.54 ± 0.0033	2.33 ± 0.0067
Z1	1.47 ± 0.0120	0.66 ± 0.0033	0.63 ± 0.0153	1.55 ± 0.0067	2.23 ± 0.0088	1.78 ± 0.0100
F2	1.64 ± 0.0232	1.56 ± 0.0451	0.59 ± 0.0384	1.76 ± 0.0265	1.05 ± 0.0260	2.15 ± 0.0333
Z2	1.52 ± 0.0548	1.14 ± 0.0867	0.39 ± 0.0296	1.63 ± 0.0273	1.35 ± 0.1100	1.88 ± 0.0960

F1 and Z1 represent the secondary trunk and lateral branch of *G. biloba* during the germination stage. F2 and Z2 represent the secondary trunk and lateral branch of *G. biloba* during the elongation stage.

### 3.3 Transcriptome sequencing

#### 3.3.1 Quality evaluation of sequencing libraries and comparison results with reference genomes

In this study, transcriptome sequencing was performed on 12 *G. biloba* samples (including secondary trunk and control of two periods, three biological replicates respectively). The results showed that the Clean Reads Rate in each sample was above 96%, and after filtering, the Clean Q30 Bases Rate was all above 92.99%. The percentage of gene sequences of each sample aligned to reference genome sequence was above 88%. The results of gene sequence alignment showed that all samples met the sequencing requirements, providing quality assurance for subsequent bioinformatics analysis (**Table 3**; **Figure S1**). The clustering results of differentially expressed genes showed that the differentially expressed genes could be roughly divided into four categories, namely, the secondary trunk (TF) and control (CF, the part without the secondary trunk) at the germination stage as well as the secondary trunk (TS) and control (CS, the part without the secondary trunk) at the elongation growth stage. All repetition groups were clustered into the same category, with good repeatability within the group (**Figure S2A**).

**Table 3:** The quality of sequencing library and comparison of cDNA library of sample and reference genome of *G. biloba*.

Sample	Clean Reads Number	Clean Reads Rate(%)	Clean Q30 Bases Rate(%)	Mapped Reads	Mapping Rate
CF1	41,474,404	97.48	93.36	37, 628, 351	0.9073
CF2	43,072,218	96.97	93.4	38, 660, 133	0.8976
CF3	47,253,398	97.61	93.74	42, 477, 701	0.8989
CS1	45, 053, 288	97.74	93.23	40, 667, 095	0.9026
CS2	47,512,890	97.66	94.05	42, 994, 997	0.9049
CS3	44,086,324	97.31	93.81	39, 409, 256	0.8939
TF1	42, 085, 858	97.48	94.33	38, 460, 000	0.9138
TF2	44, 025, 658	97.65	93.46	39, 373, 354	0.8943
TF3	38, 561, 556	97.64	93.67	34, 432, 465	0.8929
TS1	44, 387, 138	97.58	93.13	40, 114, 174	0.9037
TS2	47, 149, 178	97.83	92.99	41, 689, 486	0.8842
TS3	47, 419, 422	97.78	93.99	42, 503, 427	0.8963

TF, The repeated groups of secondary trunk at the germination stage; TS, The repeated groups of secondary trunk at the elongation stage; CF, The repeated groups of control samples around secondary trunk during germination; CS, The repeated groups of the control samples around secondary trunk during the elongation period. (1) Clean Reads Number: The total number of filtered high-quality sequences. (2) Clean Reads Rate (%): The percentage of the number high-quality sequences obtained after filtering to the number of raw sequences. (3) Clean Q30 Bases Rate (%): After filtration, the percentage of bases whose mass value is greater than 30 in the total sequence. (4) Mapped Reads: The number of sequences of each sample aligned to reference genomic. (5) Mapping Rate: The percentage of each sample sequence aligned to the reference genome sequence.

#### 3.3.2 Differential expression analysis

The statistical results of differentially expressed genes showed that a total of 74 significantly different genes were screened out in the TF vs. CF comparison group (3 up-regulated and 71 down-regulated expressions). A total of 104 significantly different genes (76 up-regulated and 28 down-regulated expressions) were screened out in the TF vs. TS comparison group. A total of 2,151 significantly different genes (1368 up-regulated and 783 down-regulated expressions) were screened out in the TS vs. CS comparison group (**Table 4**; **Figures S2B**, **C**).

**Table 4:** Number of differentially expressed genes in each comparison group of *G. biloba*.

Comparison group	Up	Down	Total
TF vs. CF	3	71	74
TF vs. TS	76	28	104
TS vs. CS	1, 368	783	2, 151

(1) Up, Up-regulated genes; (2) Down, Down-regulated genes; (3) Total, Sum of differential genes.

#### 3.3.3 GO function analysis of differentially expressed genes

All DEGs were annotated in the KEGG database, NR database, Swissprot database, KOG database, GO database, Pfam database, etc. The functional enrichment analysis of the annotated DEGs in the GO database could reflect the cell state of the bud of the secondary trunk and the control group at different stages. GO analysis was performed on the differential genes produced at two stages of secondary trunk development compared with the control group. It was found that during the process of secondary trunk development, the differential genes in the two stages were involved in the metabolic process, cell process, single organism process, cell part, membrane part, catalytic activity and protein binding in biological processes. It was speculated that the above aspects were closely related to the germination and growth of the buds of the secondary trunk (**Figures S3** –**S5**).

#### 3.3.4 KEGG pathway analysis

All differentially expressed genes were enriched into 126 KEGG pathways. In all comparison groups, pathways enriched to the top 15 in the number of differential genes were shown in **Table 5**. 42 differentially expressed genes were enriched in plant hormone signal transduction pathways (32 up-regulated and 10 down-regulated genes) (**Figure S6**; **Table 5**).

**Table 5:** Pathways enriched to the top 15 in the number of differential genes.

Pathway name	Ko	Number of up-regular DEGs	Number of down-regular DEGs	Total number
Plant hormone signal transduction	ko04075	32	10	42
Cutin, suberine and wax biosynthesis	ko00073	40	1	41
Phenylpropanoid biosynthesis	ko00940	35	4	39
Phenylalanine metabolism	ko00360	30	4	34
Plant-pathogen interaction	ko04626	23	8	31
Biosynthesis of amino acids	ko01230	22	4	26
Carbon metabolism	ko01200	22	3	25
Amino sugar and nucleotide sugar metabolism	ko00520	16	8	24
Flavonoid biosynthesis	ko00941	18	4	22
Glycerophospholipid metabolism	ko00564	18	1	19
Purine metabolism	ko00230	16	3	19
Glycolysis/Gluconeogenesis	ko00010	17	2	19
Pyruvate metabolism	ko00620	15	3	18
Starch and sucrose metabolism	ko00500	13	3	16
Glycerolipid metabolism	ko00561	15	0	15

#### 3.3.5 The regulation of the germination and development of the secondary trunk of *G. biloba*

**Figure 4:**
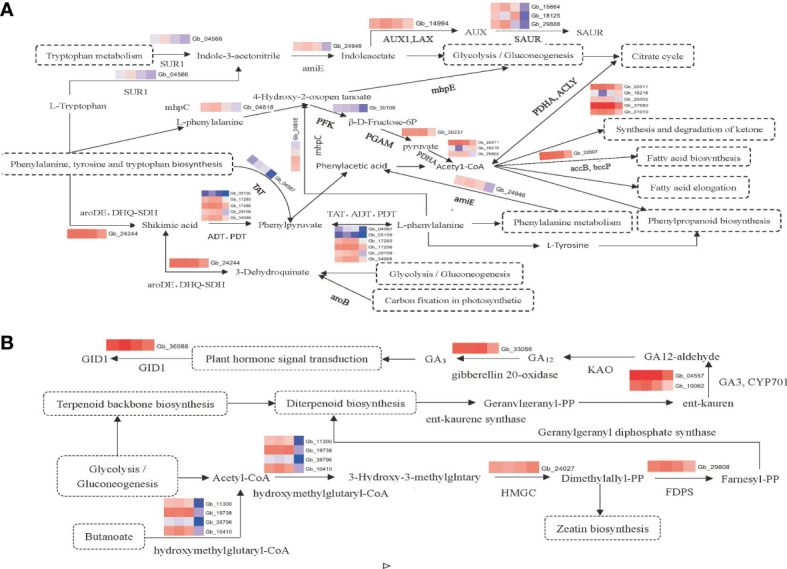
**(A)** Pathway map of differential gene expression related synthesis and regulation of IAA. **(B)** Pathway map of differential gene expression related synthesis and regulation of gibberellin.

Based on the results of endogenous hormone determination and transcriptomic data analysis, the molecular regulatory pathways related to the germination and development of the secondary trunk of *G. biloba* were plotted. The results showed that the germination and development of secondary trunk were closely related to plant hormone signal transduction, phenylpropane biosynthesis, phenylalanine metabolism, glycolysis and other metabolic pathways, and there were complex mutual regulatory relationships, among which the synthesis and regulation of IAA and GA_3_ played an important role in the development of the secondary trunk (**Figures 4A, B**).

#### 3.3.6 Verification of differential gene expression

**Figure 5:**
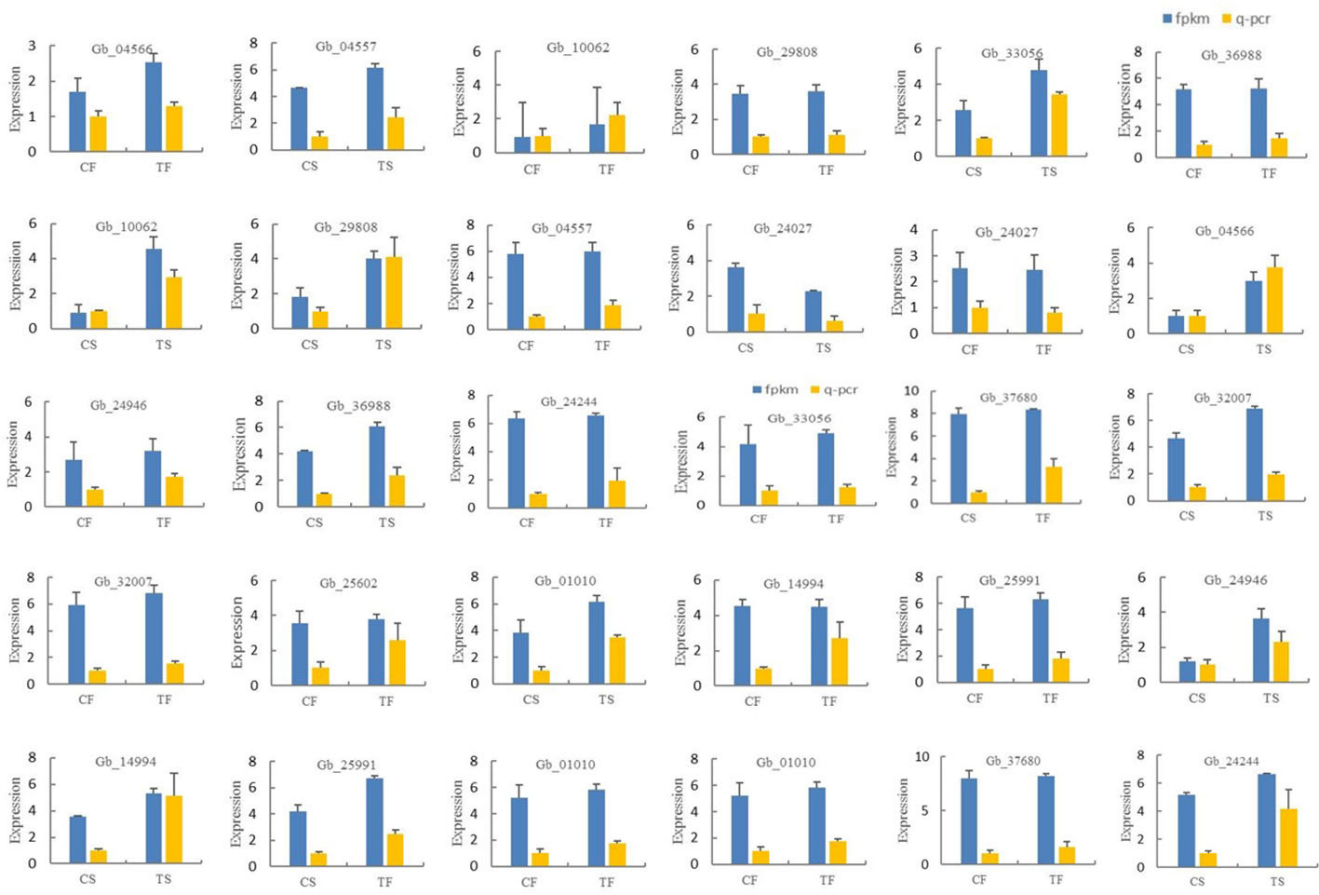
Expression verification of differentially expressed genes in different samples of *G. biloba*.

In this study, 15 differentially expressed genes were randomly selected for qPCR verification. The results showed that the expression trend of these 15 differentially expressed genes was generally consistent with the transcriptome sequencing data. *Gb36988*, *Gb33056*, *Gb10062*, *Gb29808*, *Gb04557*, *Gb04566*, *Gb24946*, *Gb14994*, *Gb25911*, *Gb01010*, *Gb25602*, *Gb32007*, *Gb37680*, and *Gb24244* were up-regulated during the two development stages of the secondary trunk and down-regulated in the control group. *Gb24027* was down-regulated in the two developmental stages of the secondary trunk and up-regulated in the control group. The results showed that the transcriptome sequencing data was accurate (**Figure 5**).

## 4 Discussion

The origin of the secondary trunk of *G. biloba* has been controversial (Fujii, 1985; Tredici, 1992b). Some studies suggested that the secondary trunk of *G. biloba* was produced by the chichi which was another special organ of *G. biloba*. Latent adventitious buds at the apex of rooted chichi could germinate into upgrowing branches under certain conditions and then developed into the secondary trunk (Xing, 1996; Fu, 2014). The chichi could produce roots and branches after it touched the ground (Fujii, 1985; Tredici, 1992a). It was also believed that the secondary trunk of *G. biloba* originated from the latent buds in the cortex of the stem at the junction of the root and stem. The secondary trunk was ‘shoots from the stem’ rather than shoots from the roots, part of the ontogenetic development of *G. biloba*, but no clear anatomical evidence has been found (Xing, 1996). This study confirmed the result and, in conjunction with previous studies, suggested that the origin of the secondary trunk should be divided into two categories. One type is produced by adventitious buds in the chichi, which can produce adventitious roots to provide nutrients for the secondary trunk, with a high germination rate. The other type is directly developed from the latent buds that exist in the cortex of the stem at the junction of the root and stem, which relies on the roots of the main stem to transport nutrients for development. The secondary trunk itself can not produce roots, retaining the characteristics of the main stem. The germination rate of the secondary trunk in the natural state is low and a large amount of germination does not occur until external stimulation. The secondary trunk of *G. biloba* may belong to autologous inhibitory dormancy, which allows plants to devote resources to controlling plant structure and reproductive growth, and allows regeneration when shoots are damaged (Horvath et al., 2003). The occurrence of the secondary trunk may be related to the age of *G. biloba*. With the increase of age, the secondary trunk will increase significantly after the senescence of the tops of the tree (Xing, 1996; Men et al., 2021). The secondary trunk is a reproductive strategy of *G. biloba* to sustain a life system when the tree reaches its growth limit or to resist bad environments and is the key to the preservation of *G. biloba* (Xiang et al., 2000; Lin and Zhang, 2004).

The secondary trunks are produced by breaking the dormancy of the dormant buds within the cortex at the junction of the root and stem of *G. biloba*, and are “branches from the stem”, but the secondary trunks are different from normal lateral branches in many aspects. The secondary trunk has obvious characteristics of “return to juvenile”, with an upright shape and a significantly smaller angle to the main stem than that of the lateral branches. Usually, the growth rate of the secondary trunk is significantly higher than that of the main stem, which can eliminate the position effect (Xing and Miao, 1996). This study found that the development of the secondary trunk was closely related to the regulation of endogenous hormones. At the germination stage, the content of each hormone in the secondary trunk was higher than that in the lateral branches, and the content of ZA and GA_3_ was significantly higher than that in the lateral branches. ZA could directly promote the growth of the lateral buds and played an important role in the germination stage of the secondary trunk (Lin and Zhang, 2004; Müller and Leyser, 2011).

GA_3_ might play a negative regulatory role in regulating the growth of lateral buds in *Arabidopsis* (Silverstone et al., 1997), rice (Qi et al., 2011) and hybrid aspen (Mauriat et al., 2011). However, for perennial *G. biloba*, the content of GA_3_ was significantly increased at the early developmental stage of the secondary trunk, and with the elongation growth, the content of IAA and ZA increased significantly and the GA_3_ content decreased significantly but was still higher than that of normal lateral branches. This is because the secondary trunk buds are in a dormancy state at the early stage of germination and the higher content of GA_3_ is conducive to breaking the dormancy and promoting germination. When the secondary trunk began to grow high, the GA_3_ gradually decreased and the content of each hormone was still higher than that of the lateral branches, indicating that the growth rate of the secondary trunk is higher than that of the lateral branches. ABA is an inhibitor of dormancy, and GA_3_ can counteract the inhibitory effect of ABA (Faust et al, 1997). GA_3_ can also promote the growth of lateral buds of papaya and mulberry (Ni, 2015). It indicates that GA_3_ is not unique to *G. biloba* in promoting the development of secondary trunk buds. Although GA_3_ has a negative regulatory effect on lateral buds in a few plants, GA_3_ promotes the growth of lateral buds for many woody plants (Ni, 2015).

The development of buds is usually closely related to the ratio of IAA to ZA in the primary tissue (Beveridge et al., 2003). At the germination stage, the (IAA+ZA+GA_3_)/ABA value of the secondary trunk and lateral branches were greater than 1, and the value of the secondary trunk was significantly greater than that of the lateral branches. It indicates that they are all growing and the growth trend of the secondary trunk is more obvious than that of lateral branches. The smaller IAA/ZA value is more favorable to the germination of the secondary trunk. In the elongation growth period, the (IAA+ZA+GA_3_)/ABA value of the secondary trunk decreased compared with the germination period and the growth trend slowed down, while the ratio of lateral branches increased slightly, which was due to the difference in the time between the secondary trunk and lateral branches entering the rapid growth period. The secondary trunk responds quickly to internal and external environmental signals, while the lateral branches respond relatively slowly.

Through transcriptome analysis, the GO enrichment analysis of the screened differentially expressed genes revealed that the differentially expressed genes related to the secondary trunk development were mainly enriched in the metabolic process, cell process, cell part, membrane part, catalytic activity and protein binding in the biological processes. It was generally consistent with the research results of Yang Lili about the release of natural dormancy of grape winter buds (Yang et al., 2020).

The secondary trunk is a unique organ of *G. biloba*. The dormancy of secondary trunk buds is controlled by the environment and genes. Combined with the endogenous hormone determination and sequencing analysis, it was found that endogenous hormones may be an important internal cause for the development of the secondary trunk. The development of the secondary trunk is closely related to several metabolic pathways and has complex interrelationships, especially with the synthesis and metabolism of hormones. The genes related to phenylalanine metabolism and phenylpropane biosynthesis were significantly enriched at the early developmental stage of adventitious bud in *Arabidopsis*, and phenylalanine significantly inhibited the development of adventitious bud in *Arabidopsis* (Wang et al., 2013).

However, these two pathways were also significantly enriched in this study. Several genes related to the biosynthetic pathways of phenylalanine, tyrosine and tryptophan were enriched in pathways related to the synthesis and regulation of IAA. For example, genes related to L-phenylalanine synthesis, such as *Gb24244*(*aroDE*, *DHQ-SDH*), *Gb00109*(*ADT*, *PDT*), *Gb17285*(*ADT*, *PDT*), *Gb17286*(*ADT*, *PDT*), *Gb29709*(*ADT*, *PDT*), *Gb34068*(*ADT*, *PDT*), *Gb04567*(*TAT*), were up-regulated in the secondary trunk group. The synthesis of shikimic acid could be promoted by *DHQ-SDH*, and then L-phenylalanine could be synthesized by tyrosine transaminase (*TAT*) and aromatic acid dehydrase (*ADT*, *PDT*), which inhibited the germination of the secondary trunk at the initial stage of germination. Phenylalanine was involved in phenylpropane biosynthesis and phenylalanine metabolism. In addition, the tryptophan metabolism-related genes *Gb04566*(*SUR1*)and *Gb24946*(*amiE*), and IAA signal response-related genes *Gb14994*(*AUX1*), *Gb15664*(*SAUR*), and *Gb29888*(*SAUR*) in plant hormone signal transduction pathway were also enriched, all up-regulated in the secondary trunk group. Tryptophan is the substrate of IAA synthesis. When *G. biloba* is stimulated by the certain internal and external environment, the up-regulated expression of *Gb04566* (*SUR1*) promotes the accumulation of indole-3-acetonitrile, which increases the content of indole-3-acetic acid and upregulates the expression of IAA inflow vector gene in the tryptophan metabolic pathway. Finally, the IAA response gene SAUR receives and responds to the IAA signal to stimulate the development of the secondary trunk. In addition, the biosynthetic pathway of phenylalanine, tyrosine, and tryptophan can also produce L-tryptophan, which facilitates the accumulation of indole-3-acetonitrile and thus the synthesis of indole-3-acetic acid. At the same time, indole-3-acetic acid can indirectly promote the glycolysis pathway, which uses starch and sucrose metabolism as substrates to provide energy for the development of the secondary trunk.

The biosynthesis pathways of phenylalanine, tyrosine and tryptophan are important pathways linking tryptophan and phenylalanine, promoting the synthesis of acetyl-CoA through the intermediate products such as phenylpyruvate, phenylacetic acid, phenylalanine, and 4-hydroxy-2-oxy valerate. Acetyl-CoA is involved in phenylalanine metabolism, ketone body biosynthesis, phenylpropane biosynthesis, fatty acid synthesis and fatty acid prolongation. In addition, the carbon fixation pathway in glycolysis and photosynthetic organisms can also produce phenylalanine through the shikimic acid pathway (Sun, 2019).

GA_3_ can break the dormancy of buds and promote secondary trunk germination (Mäkilä et al., 2023), and the same results were obtained in the transcriptome. Genes related to gibberellin synthesis including *Gb33056*(gibberellin 20-oxidase), *Gb04557*(*GA_3_*, *CYP701*)and *Gb10062*(*GA_3_*, *CYP701*), and genes related to plant hormone signal transduction including *Gb36988*(*GID1*)and genes promoting diterpenoid biosynthesis including *Gb11300*, *Gb19738*, *Gb39796*, *Gb10410*, *Gb24027*(*HMGCR*) and *Gb29808*(*FDPS*) were enriched in the diterpenoid biosynthesis pathway. The terpene skeleton biosynthesis pathway is based on glycolysis to promote diterpene biosynthesis. Firstly, acetyl-CoA is synthesized, and the synthesis of isoamyl diphosphate is promoted by hydroxy methyl glutaryl-CoA synthase and hydroxy methyl glutaryl-CoA reductase (*HMGCR*). And then Farnesyl pyrophosphate synthase (*FDPS*) which is the key enzyme related to diterpenoids synthesis promotes the synthesis of farnesyl pyrophosphate and further promotes the diterpenoid biosynthetic pathway. In the diterpene biosynthesis pathway, the terpene skeleton biosynthesis is used as the substrate to synthesize geranylgeranyl pyrophosphate which is the substrate of gibberellin to further synthesized kaurene. Through the up-regulated expression of *Gb04557* and *Gb10062*, kaurene oxidase (*GA_3_*, *CYP701*) promotes the synthesis of GA_12_ aldehyde and further synthesizes various gibberellins, including GA_3_. The accumulation of gibberellin can promote the phytohormone signal transduction pathway to up-regulate the expression of gibberellin receptor *GID1* (*Gb36988*). And it can break the dormancy of secondary trunk buds in the cortex of the *G. biloba* stem and promote the germination and growth of the secondary trunk. In addition, isoamyl diphosphate, an intermediate product in the terpene skeleton biosynthetic pathway, can also promote zeatin biosynthesis and may play a role in the development of the secondary trunk.

The secondary trunk may be a reproductive strategy of *G. biloba* to sustain the life system when *G. biloba* reached its growth limit or to resist the adverse environment. It is a natural clonal multi-generation reproduction phenomenon. *G. biloba* can continuously produce new secondary trunks to make the mother tree that has reached its life limit rejuvenate and improve adaptability. Reproduction and renewal through secondary trunks is the key to the survival of *G. biloba* after catastrophes (Xiang et al., 2000; Fu et al., 2013).

## 5 Conclusions

The development process of secondary trunk of *G. biloba* can be divided into four stages, namely, dormancy stage, differentiation stage, conduction tissue formation stage, and germination stage. The contents of IAA, ZA, and GA_3_ in the budding stage of secondary trunk development were higher than those in the lateral branches, breaking dormancy of dormant buds and promoting secondary trunk germination. After the germination stage, the content of GA_3_ significantly decreased, while the content of IAA and ZA significantly increased, and the secondary trunk entered the elongation growth stage. In secondary trunk, the ratio of (IAA+ZA+GA_3_)/ABA is greater than that of lateral branches, while the ratio of IAA/ZA is extremely smaller than that of lateral branches, so the growth speed of secondary trunk is higher than that of lateral branches. Through transcriptome sequencing, the differentially expressed genes were screened out, and the key regulatory pathways for the occurrence and development of secondary trunk was sorted out. Phenylalanine metabolism and phenylpropane biosynthesis may inhibit the germination of early secondary trunk buds. After being stimulated by the certain internal and external environment, *G. biloba* synthesizes gibberellin using terpene skeleton biosynthesis as the substrate, which can promote the plant hormones signal transduction pathway and upregulate the expression of gibberellin receptor (*GID1*) to break the dormancy state of secondary trunk buds. At the same time, genes related to IAA synthesis are upregulated and indole-3-acetic acid content is increased, leading to the up-regulated expression of IAA intracellular vector genes. The IAA response gene (*SAUR*) receives and responds to IAA signals to promote the development of the secondary trunk.

## Data availability statement

The data presented in the study are deposited in the NCBI Sequence Read Archive (SRA), accession numbers SRR23730290, SRR23730291, SRR23730292, SRR23730293, SRR23730294, SRR23730288, SRR23730289, SRR23730295, SRR23730296, SRR23730297, SRR23730298, SRR23730299.

## Author contributions

L-MS designed the research; Z-YC and L-MS carried out the experiments; Z-YC and L-NS analyzed the data and wrote the manuscript; L-NS revised the manuscript. Conceptualization, L-MS. Writing—original draft preparation, Z-YC and L-NS. Writing—review and editing, L-MS. Data curation, X-YZ and X-JK. Experiments preformation, Z-YC, L-NS, QZ, and X-LH. Supervision, funding acquisition, and project administration, L-MS. All authors contributed to the article and approved the submitted version.

## Supplementary material

Supplementary Figure 1 Q30 quality control chart.

Supplementary Figure 2
**(A)** Cluster Map of DEGs. **(B)** Statistics of differentially expressed genes in each comparison group of *G. biloba*. **(C)** Volcano map of DEGs in each comparison group of *G. biloba.* The abscissa is the expression multiple, and the ordinate is the significance degree of the change of expression quantity (P <0.05). Orange indicates genes that are significantly up-regulated in differential genes. Green indicates genes that are significantly down-regulated, and gray indicates genes that are not significantly different.

Supplementary Figure 3 Distribution of q-values for enriched GO entries in each comparison group of *G. biloba*. Take the enriched GO entries in all samples for analysis, the ordinate is the GO entry and the abscissa is the name of different comparison groups. Different colors represent different degrees of enrichment.

Supplementary Figure 4 Histogram of GO statistics of differentially expressed genes in each comparison group of *G. biloba*. The abscissa is each major category under GO, which represents various biological processes, cell components and molecular functions. The left ordinate is the proportion of this category, and the right ordinate is the specific number of genes in this category. Different colors represent different groups (differentially expressed genes are up-regulated and down-regulated).

Supplementary Figure 5 Q-value enrichment chart of GO items in each comparison group of *G. biloba.* GO items enriched in all comparison groups were taken for analysis, and Q-value enrichment analysis was conducted for the three categories respectively. The ordinate is the secondary entry of GO and the abscissa is the degree of enrichment. Each point represents the degree of enrichment of the GO entry. The closer the color is to red, the higher the degree of enrichment. The size of each point indicates the number of genes enriched in the GO entry. The larger the point, the more genes are enriched in the GO entry, and vice versa.

Supplementary Figure 6 Distribution of q-values of enrichment pathways in each comparison group of *G. biloba*.
