# Supplementary figures and images for: The development and transcriptome regulation of the secondary trunk of Ginkgo biloba L
Source: Front Plant Sci. 2023 May 30;14:1161693. doi: 10.3389/fpls.2023.1161693 (PMC10267747; doi:10.3389/fpls.2023.1161693)

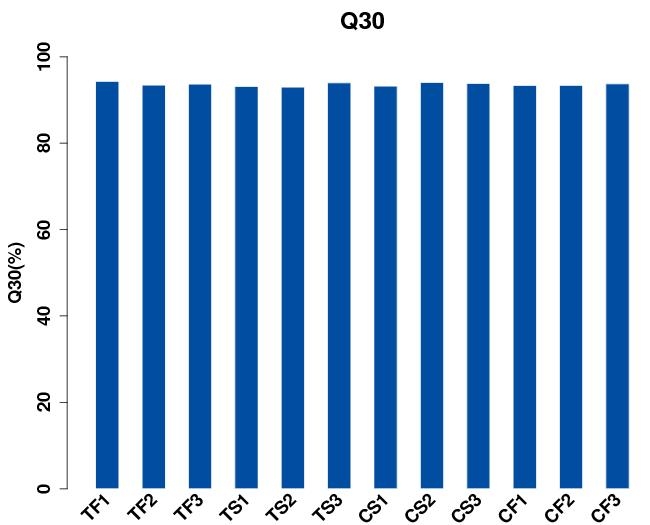

Supplement: Supplementary Figure 1 — Q30 quality control chart. [file Image_1.jpeg]

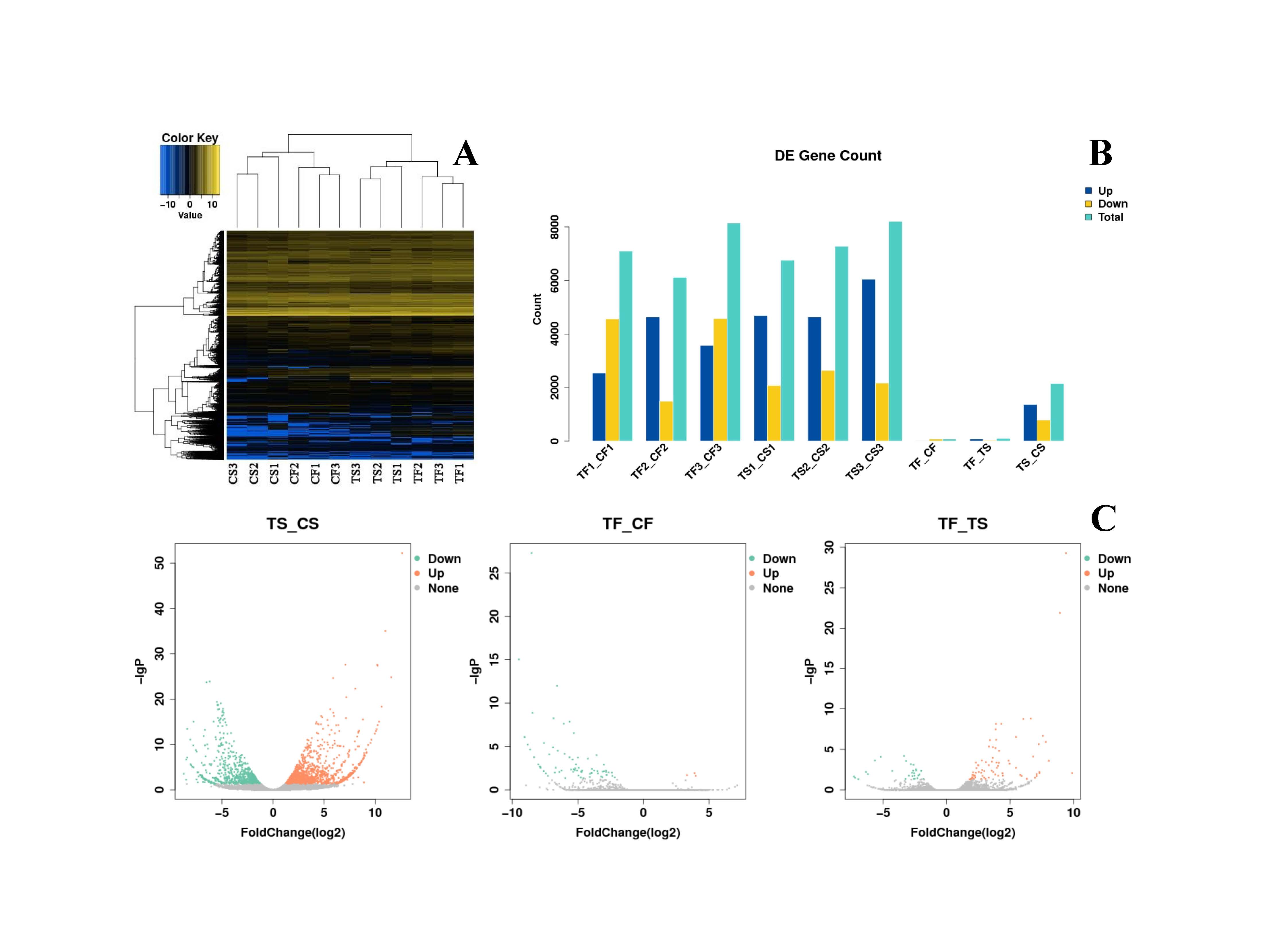

Supplement: Supplementary Figure 2 — (A) Cluster Map of DEGs. (B) Statistics of differentially expressed genes in each comparison group of G. biloba. (C) Volcano map of DEGs in each comparison group of G. biloba. The abscissa is the expression multiple, and the ordinate is the significance degree of the change of expression quantity (P <0.05). Orange indicates genes that are significantly up-regulated in differential genes. Green indicates genes that are significantly down-regulated, and gray indicates genes that are not significantly different. [file Image_2.jpeg]

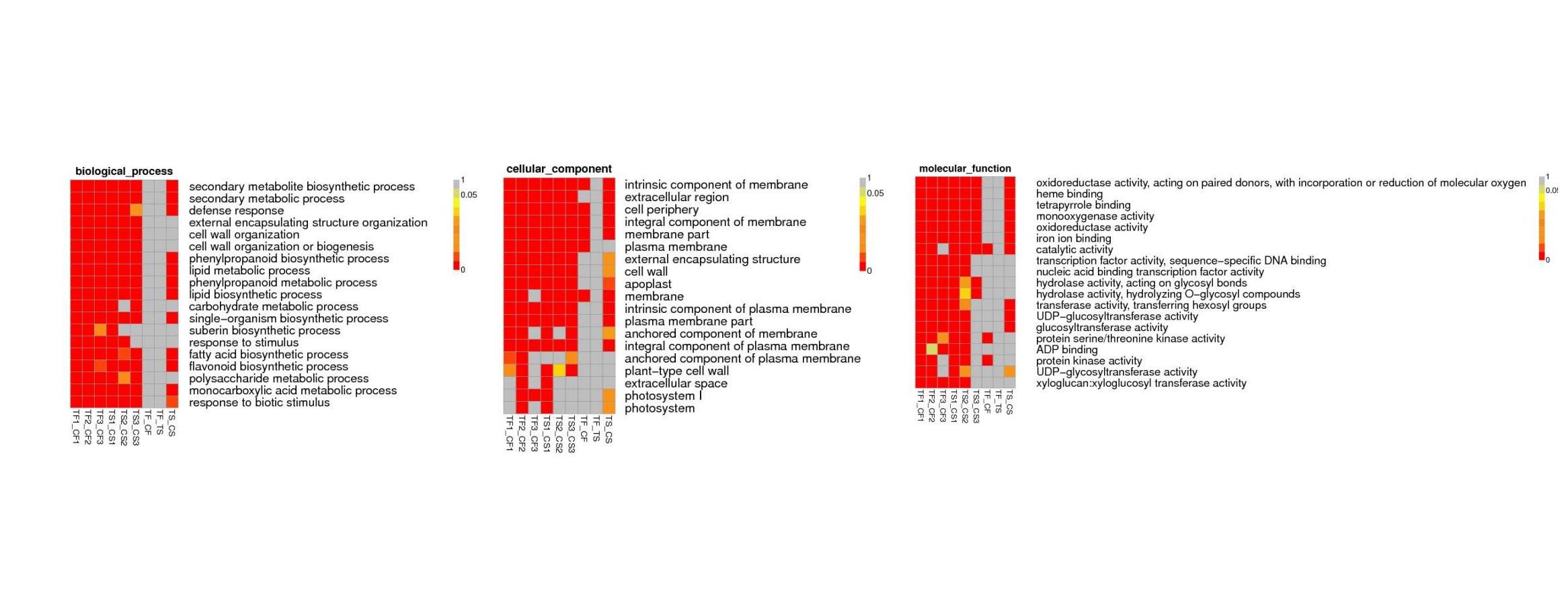

Supplement: Supplementary Figure 3 — Distribution of q-values for enriched GO entries in each comparison group of G. biloba. Take the enriched GO entries in all samples for analysis, the ordinate is the GO entry and the abscissa is the name of different comparison groups. Different colors represent different degrees of enrichment. [file Image_3.jpeg]

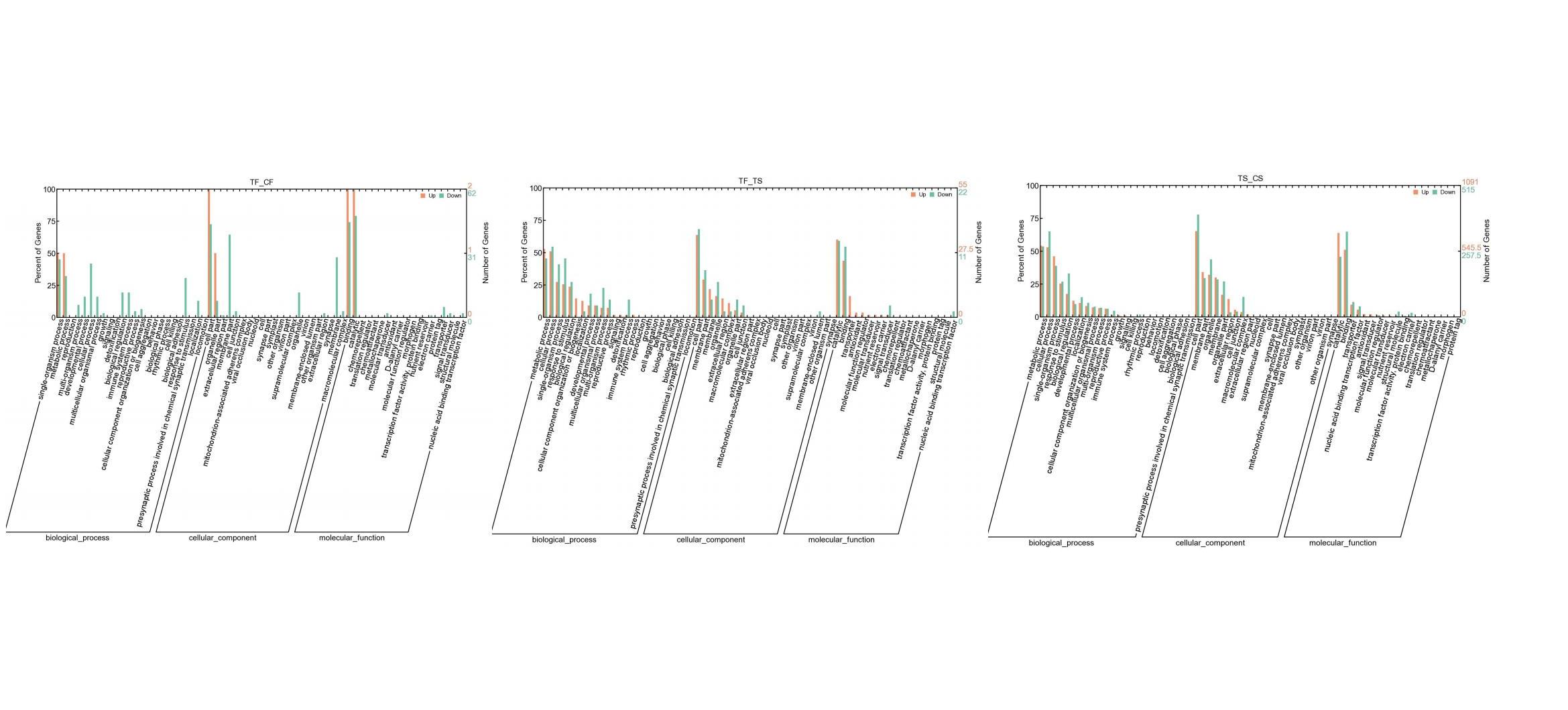

Supplement: Supplementary Figure 4 — Histogram of GO statistics of differentially expressed genes in each comparison group of G. biloba. The abscissa is each major category under GO, which represents various biological processes, cell components and molecular functions. The left ordinate is the proportion of this category, and the right ordinate is the specific number of genes in this category. Different colors represent different groups (differentially expressed genes are up-regulated and down-regulated). [file Image_4.jpeg]

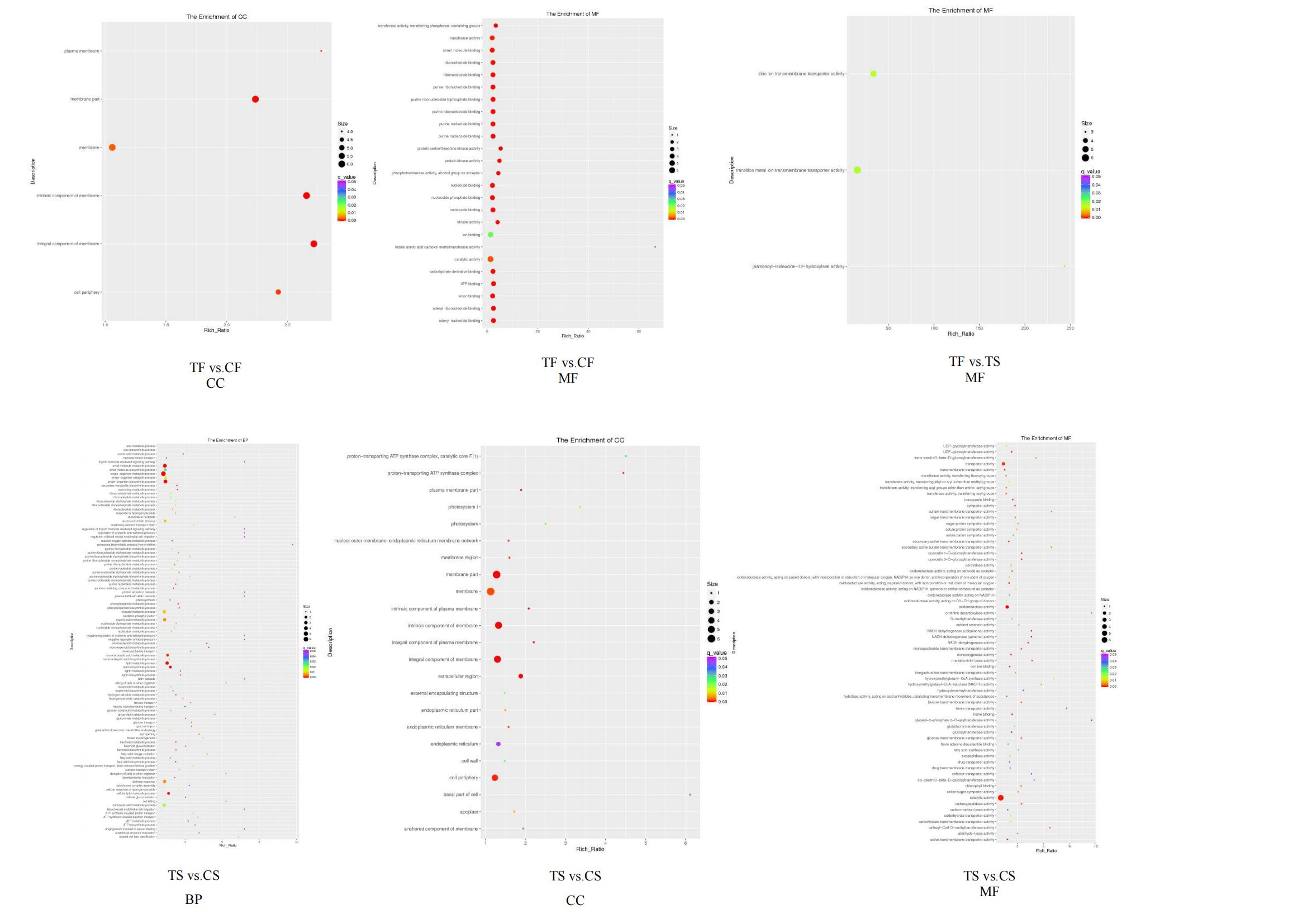

Supplement: Supplementary Figure 5 — Q-value enrichment chart of GO items in each comparison group of G. biloba. GO items enriched in all comparison groups were taken for analysis, and Q-value enrichment analysis was conducted for the three categories respectively. The ordinate is the secondary entry of GO and the abscissa is the degree of enrichment. Each point represents the degree of enrichment of the GO entry. The closer the color is to red, the higher the degree of enrichment. The size of each point indicates the number of genes enriched in the GO entry. The larger the point, the more genes are enriched in the GO entry, and vice versa. [file Image_5.jpeg]

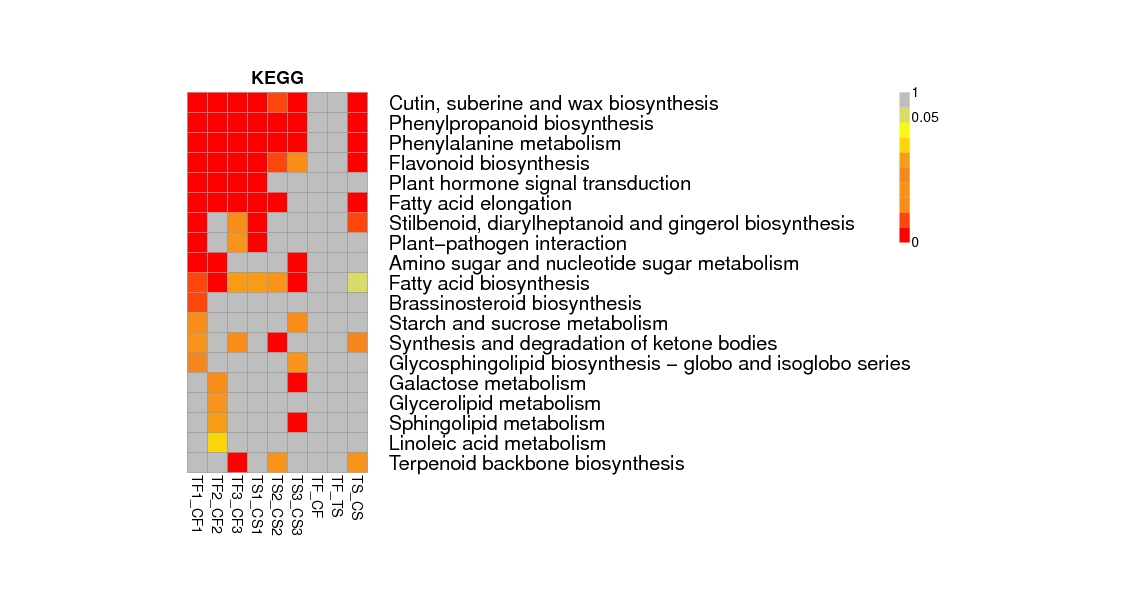

Supplement: Supplementary Figure 6 — Distribution of q-values of enrichment pathways in each comparison group of G. biloba. [file Image_6.jpeg]
